# Comparison of the effect of lps and pam3 on ventilated lungs

**DOI:** 10.1186/1471-2466-10-20

**Published:** 2010-04-20

**Authors:** Hans P Hauber, Dörte Karp, Torsten Goldmann, Ekkehard Vollmer, Peter Zabel

**Affiliations:** 1 Pathophysiology of Inflammation, Research Center Borstel, Borstel, Germany; 2 Medical Clinic, Research Center Borstel, Borstel, Germany; 3 Experimental Pathology, Research Center Borstel, Borstel, Germany

## Abstract

**Background:**

While lipopolysaccharide (LPS) from Gram-negative bacteria has been shown to augment inflammation in ventilated lungs information on the effect of Gram-positive bacteria is lacking. Therefore the effect of LPS and a lipopetide from Gram-positive bacteria, PAM3, on ventilated lungs were investigated.

**Methods:**

C57/Bl6 mice were mechanically ventilated. Sterile saline (sham) and different concentrations of LPS (1 μg and 5 μg) and PAM3 (50 nM and 200 nM) were applied intratracheally. Lung function parameters and expression of MIP-2 and TNFα as well as influx of neutrophils were measured.

**Results:**

Mechanical ventilation increased resistance and decreased compliance over time. PAM3 but not LPS significantly increased resistance compared to sham challenge (P < 0.05). Both LPS and PAM3 significantly increased MIP-2 and TNFα mRNA expression compared to sham challenge (P < 0.05). The numbers of neutrophils were significantly increased after LPS at a concentration of 5 μg compared to sham (P < 0.05). PAM3 significantly increased the numbers of neutrophils at both concentrations compared to sham (P < 0.05).

**Conclusions:**

These data suggest that PAM3 similar to LPS enhances ventilator-induced inflammation. Moreover, PAM3 but not LPS increases pulmonary resistance in ventilated lungs. Further studies are warranted to define the role of lipopetides in ventilator-associated lung injury.

## Background

Invasive mechanical ventilation is a life saving approach in severe respiratory failure. However, large tidal, high inspiratory pressures, and end-exspiratory alveolar collapse with cyclic reopening can damage the lung parenchyma leading to ventilator-induced lung injury (VILI) [1-6]. Several studies have provided insight into the pathogenesis of VILI and the underlying inflammatory processes (reviewed in [7]).

One major problem in mechanically ventilated patients is ventilator-associated pneumonia (VAP). Incidence rates in the intensive care unit (ICU) vary from 8% to 28% [8]. Mortality rates can reach 76% depending on high-risk pathogens [8]. A number of potential causative factors have been identified (e. g. orotracheal tube, impaired mucociliary clearance, severely ill patients, multiresistant bacteria) [9]. In addition, previous studies have demonstrated that bacterial products such as lipopolysaccharide (LPS) from Gram-negative bacteria augment ventilator-induced inflammation [10-12]. Most of that previous work has focussed on expression of inflammatory mediators and inflammatory cells. Changes in lung function parameters have been less well characterized. Moreover, LPS has been administered systemically or intratracheally before the beginning of ventilation [10-12].

In contrast to LPS, data on the effect of Gram-positive bacteria on inflammation and lung function in ventilated lungs are lacking despite the importance of Gram-positive bacteria in VAP [8]. Therefore we sought to compare the functional and structural effects of intratracheal application of Gram-negative and Gram-positive bacterial products to ventilated lungs in a mouse model of mechanical ventilation. We chose to compare the effect of LPS to a lipopetide form the outer cell membrane of bacteria, PAM3.

## Methods

### Animal preparation

Experiments were carried out in accordance with the Animal Protection Law of Germany. All experiments were approved by the local Ethics Committee. 10- to 12-week old male C57/Bl6 mice (Charles River Labs, Berlin, Germany) (25-35 g) were anesthetized by intraperitoneal injection of ketamine (100 mg/kg) and xylazin (20 mg/kg). Additional anesthetic was given when animals start to gain consciousness as assessed by positive testing of paw reflex. Body temperature was maintained with a homeothermic blanket system with flexible probe (Harvard Apparatus, Holliston, USA). After tracheostomy with a secured 18-gauge metal cannula mechanical ventilation was initiated using a flexivent (Scireq, Montreal, Canada) computer-controlled small animal ventilator. Oxygen saturation and pulse rate were monitored using the MouseOx oximeter (Starr Life Science, Pittburgh, PA). The sensor was placed on the back leg along the leg axis. The mice were covered throughout the experiments to maintain body temperature.

### Protocols

After anesthesia mice were randomized to different groups. All mice were mechanically ventilated with a tidal volume (Vt) of 10 ml/kg and a positive end-expiratory pressure (PEEP) of 2 cm H2O. Breath rate was 120/min. Inspiratory fraction of oxygen was 0.21 (normal air). 9 mice were only ventilated without any challenge (control group). Intratracheal application was performed 30 min after start of ventilation with the nebulizer system form flexivent (Ana-B1/8, Scireq, Montreal, Canada) because after 30 min a plateau was reached for resistance. The total volume of solution was 50 μL (in 10 sec) in every condition. Challenges included sterile saline (sham, N = 5), 1 μg LPS (N = 6), 5 μg LPS (N = 8), 50 nM PAM3 (N = 5), and 200 nM PAM3 (N = 6). After 120 min of ventilation animals were sacrificed. At the end of each experiment the lung of each animal was divided into two parts. One part was immediately snap frozen in liquid nitrogen for later RNA extraction. The other part was put into formalin for later histological examination.

### Lung function measurements

At the beginning, every 15 min and at the end of mechanical ventilation a TLC (total lung capacity) manoeuvre was performed. With this manoeuvre lungs are inflated with a pressure of up to 30 cm H_2_O for a total of six seconds in order to standardize the volume history and to prevent pre-existing atelectasis. During mechanical ventilation lung function measurements were performed every 5 min using the flexivent ventilator. Resistance and compliance were determined with forced manoeuvres (volume of approximately 200 μl) on the basis of a single compartment model of the lungs as described previously [13]. Newtonian resistance, tissue damping and elastance were obtained using the forced oscillation technique that fits the constant-phase model to input impedence [13]. Briefly, this perturbation involves an 8-s signal, which utilizes a range of frequencies, including 0.5, 0.75, 1.25, 1.75, 2.75, 3.25, 4.25, 4.75, 5.75, 7.25, 9.25, 10.25, 11.75, 14.75, 16.75, 18.25, and 19.75 Hz.

### Histological measurements

Prior to fixation lungs were inflated until total lung capacity. Formalin fixed tissue specimens were sectioned and stained with hematoxilin eosin (HE) according to standard protocols. Numbers of polymorphonuclear leukocytes (PMN) were counted per high powered field (0.25 mm^2^) by two independent observers in a blinded fashion. The within observer coefficient of variation was less than 5%.

### RNA extraction and reverse transcription

RNA from whole lung tissue samples was extracted using an RNeasy Mini Kit (Qiagen). Reverse transcription was performed with 0.5-1.0 μg of RNA per reaction using Superscript II reverse transcriptase (RT, 200 U per reaction) (Invitrogen) and oligo-dT in the presence of an RNase inhibitor (RNase Out, Invitrogen). The RNA was reverse transcribed in 30 μl of total volume at 65°C for 10 min, at 42°C for 60 min, and at 100°C for 1 min. The resultant first-strand complementary DNA (cDNA) was used as template for PCR.

### Quantitative real time PCR (QRTPCR)

QRTPCR was carried out using a LightCycler system (Roche Diagnostics, Mannheim, Germany). Macrophage inflammatory protein (MIP)-2 mRNA, tumour necrosis factor (TNF)α mRNA and hypoxanthine phosphoribosyltransferase (HPRT) mRNA expression was quantified using QRTPCR. MIP-2 and TNFα were selected as pro-inflammatory cytokines with well known up-regulation in VILI and after LPS challenge. HPRT was used as house keeping gene. Primers were based on published mRNA sequences and were designed to span at least two exons in order to avoid binding to genomic DNA. Specific amplification using these primers was confirmed by ethidium bromide staining of the predicted size of the PCR products on an agarose gel. PCR was performed using the QuantiTect SYBR Green PCR Kit (Qiagen) with the appropriate primers and samples according to the manufacturer's protocol. In brief, 1 μl of cDNA was added to 10 μl of 2× QuantiTect SYBR Green PCR master mix, 8 μl of RNase-free water, and 0.5 μl of each primer (20 μM) resulting in a total volume of 20 μl. All PCR experiments were carried out in triplicate.

### Statistics

Changes in lung function parameters were assessed by comparison of the values at the time of challenge (30 min after the beginning of ventilation) and the values at the end of the experiment (120 min). For comparison of values at the beginning and at the end of the experiment in one group a paired t test was used. For comparison between different groups an overall ANOVA on ranks, followed by multiple testing with the Bonferroni correction, was performed. Differences between groups were assessed by means of post hoc pairwise comparison with the Dunnet test (Systat version 7.0, SPSS Inc, USA). A P value of less than 0.05 was considered statistically significant. All values are given as means ± SD if not otherwise stated.

## Results

### Effect of LPS and PAM3 on lung function parameters

**Figure 1:**
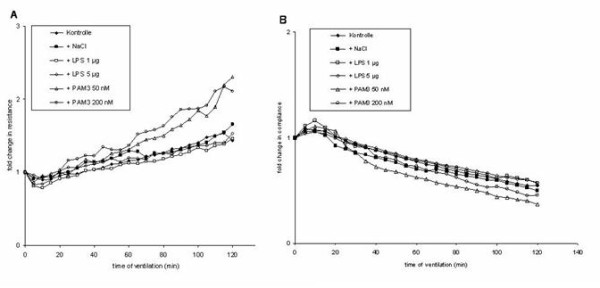
**Relative changes in resistance (A) and compliance (B) during mechanical ventilation alone and after challenge with sterile saline (sham), LPS (1 μg and 5 μg) and PAM3 (50 nM and 200 nM)**. Mean values are shown.

**Figure 2:**
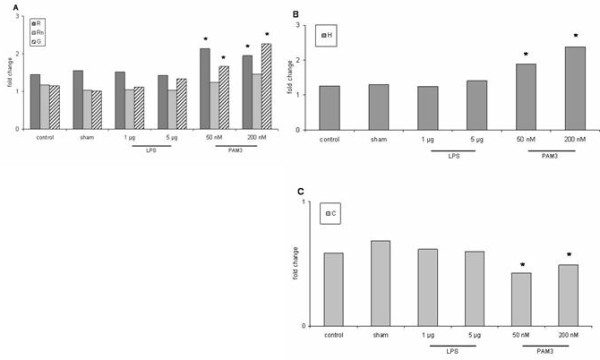
**Relative changes of lung function parameters from time of stimulation (30 min) to the end of ventilation (120 min)**. A: Resistance (R) and tissue damping (G) significantly increased after PAM3 (P < 0.05) but not in the other groups. No significant change was observed for Newtonian resistance (Rn) (P > 0.05). B: Elastance (H) significantly increased with PAM3 (P < 0.05). C: Compliance (C) was significantly decreased after stimulation with PAM3 (P < 0.05).

Resistance increased during mechanical ventilation in all groups (Figure 1A). The strongest increase was observed with PAM3. PAM3 at concentrations of 50 nM and 200 nM significantly increased total resistance (R; 2.1-fold and 1.9-fold, respectively) and tissue damping (G; 1.7-fold and 2.3-fold) from the time of challenge to the end of the experiment (P < 0.05) (Figure 2A). No significant effect was observed with Newtonian resistance (Rn) (Figure 2A). Sham challenge and LPS challenge had no significant effects on these parameters (P > 0.05) (Figure 1A and 2A).

Stimulation with PAM3 also significantly increased elastance (1.9-fold and 2.4-fold, respectively) (P < 0.05). No significant effect was noted in the other groups (P > 0.05) (Figure 2B). Compliance decreased during mechanical ventilation in all groups (Figure 1B). PAM3 led to a significant decrease (60% and 40%, respectively) (P < 0.05) whereas no significant reduction was observed in the other groups (Figure 2C).

### Effect of LPS and PAM3 on oxygen saturation and heart rate

There was no statistically significant difference in oxygen saturation and heart rate between the control group, the sham group and the challenge groups (data not shown) (P > 0.05).

### Effect of LPS and PAM3 on pro-inflammatory cytokine expression

**Figure 3:**
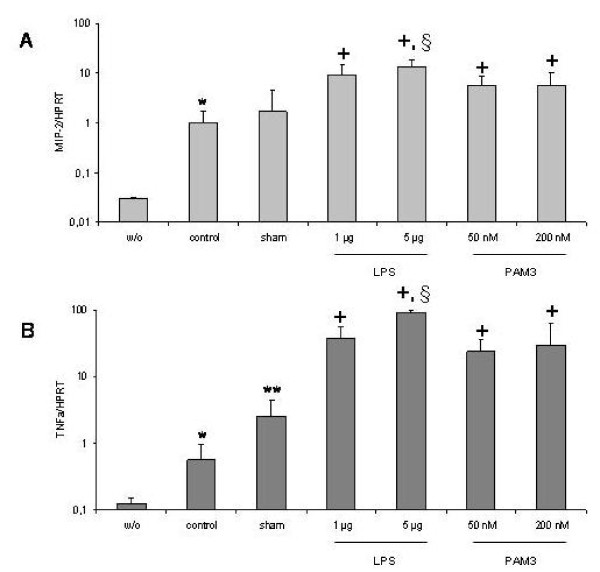
**MIP-2 (A) and TNFα (B) gene expression in the mouse lung of spontaneously breathing animals (w/o, without mechanical ventilation) and mechanical ventilated animals without challenge (control), with intratracheal saline challenge (sham) and with challenge with LPS or PAM3 at different doses**. Bars indicate mean+SEM. *: P < 0.05 vs spontaneously breathing mice. **: P < 0.05 vs control. +: P < 0.05 vs sham. § P < 0.05 vs PAM3 challenge.

Mechanical ventilation alone significantly increased MIP-2 and TNFα mRNA expression in the lungs compared to spontaneously breathing controls (33.3-fold and 5.7-fold, respectively; P < 0.05). Intratracheal challenge with NaCl (sham) further increased cytokine expression. This effect was significant for TNFα (4.4-fold; P < 0.05) but not for MIP-2 (1.7-fold; P > 0.05) compared to ventilation alone. LPS significantly increased MIP-2 (5.5-fold and 7.9-fold, respectively; P < 0.05) and TNFα mRNA expression (15.1-fold and 36.3-fold, respectively; P < 0.05) in a dose dependent manner compared to sham challenge. PAM3 also significantly increased MIP-2 (3.3-fold and 3.2-fold, respectively; P < 0.05) and TNFα mRNA expression (9.4-fold and 11.8-fold, respectively; P < 0.05) compared to sham challenge. No dose dependency was observed with PAM3. LPS had a stronger effect on cytokine expression compared to PAM3 reaching statistical significance at a dose of 5 ng/mL (P < 0.05) (Figure 3A and 3B).

### Effect of LPS and PAM3 on neutrophil inflammation in ventilated lungs

**Figure 4:**
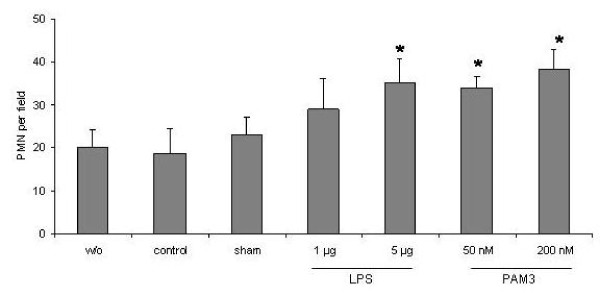
**Numbers of polymorphonuclear cells (PMN) per high powered field in the mouse lung of spontaneously breathing animals (w/o, without mechanical ventilation) and mechanical ventilated animals without challenge (control), with intratracheal saline challenge (sham) and with challenge with LPS or PAM3 at different doses**. Bars indicate mean+SEM. *: P < 0.05 vs sham.

**Figure 5:**
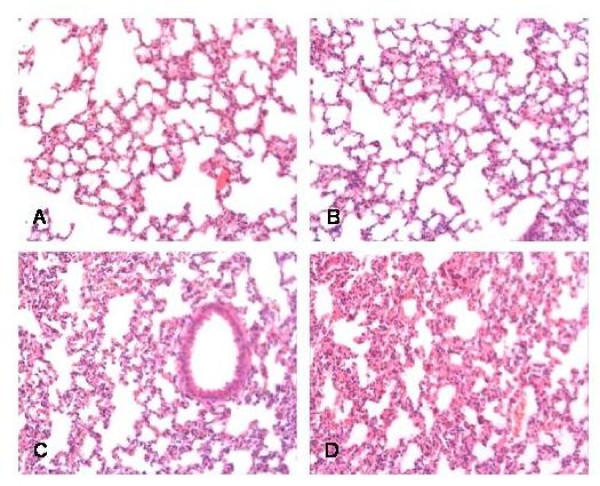
**Histology sections of mouse lungs with ventilation alone (A), after sham challenge (B), after LPS challenge (5 μg) (C), and after PAM3 challenge (200 nM) (D)**. HE staining. Original magnification ×100.

Ventilation alone as well as sham challenge had no significant effect on the numbers of neutrophils in the lungs compared to spontaneously breathing animals (23.0 ± 2.1/field and 18.6 ± 2.2/field vs 20.0 ± 2.5/field) (P > 0.05). LPS increased neutrophil numbers in a dose dependent manner. This effect was significant at a dose of 5 μg (35.0 ± 4.0/field) compared to sham (P < 0.05). PAM3 also significantly increased numbers of neutrophils being significant at both tested concentrations (34.0 ± 1.5/field and 38.3 ± 2.6/field) (P < 0.05) (Figure 4 and 5).

## Discussion

In the present study we investigated the effects of inhalation of Gram-negative and Gram-positive bacterial products on mechanically ventilated lungs. We found that both LPS and PAM3 augmented lung inflammation. Moreover, PAM3 but not LPS led to a significant increase in pulmonary resistance.

Previous studies have demonstrated inflammation due to mechanical ventilation [7,14]. In those studies mostly high tidal volume ventilation was compared to low tidal ventilation (protective ventilation) [15,16]. Several reports have demonstrated that LPS can further increase VILI or can augment inflammation in lungs receiving a protective ventilation strategy. However, in those studies LPS was applied before beginning of ventilation or systemically [10-12]. Data on the effect of LPS application on already ventilated lungs are sparse. Therefore we challenged ventilated animals with LPS. Agreeing with previous data we found increased inflammation when LPS was added to ventilated lungs.

PAM3 is a lipopetide from bacteria that binds to TLR-2 [17]. Although up-regulation of TLR-2 by mechanical ventilation has been reported previously data on the effect of PAM3 on ventilated lungs are sparse [18]. In the present study PAM3 enhanced ventilator-associated inflammation similar to LPS. However, the effect of LPS on pro-inflammatory cytokine expression and neutrophil influx was stronger compared to PAM3. We did not measure protein levels of cytokines in bronchoalveolar lavage fluid but previous studies have shown increased cytokine protein levels after LPS challenge [10]. It is not very likely that there may be difference between mRNA and protein expression after PAM3 challenge. However, this has to be considered as a limitation of our study.

We did not challenge unventilated mice with LPS or PAM3. Therefore we can only compare the effect of LPS and PAM3 in ventilated mice. However, both LPS and PAM3 further enhanced inflammation in ventilated lungs compared to sham challenge.

In the present study we show for the first time that a TLR-2 agonist, PAM3, is able to augment ventilator-associated inflammation. Recent data from the literature have questioned the role of TLR-2 in ventilator-induced inflammation [18]. Our results do not necessarily support a role for TLR-2 in the pathogenesis of VILI but they show that TLR-2 activation may be important in enhancing ventilator-induced inflammation. This finding is supported by clinical practice since VAP caused by Gram-positive bacteria can be observed frequently [10]. However, our study was not designed to investigate susceptibility to infection in ventilated lungs. The role of TLR-2 in this setting remains to be further evaluated.

Interestingly in our study PAM3 challenge led to a significant increase in resistance compared to LPS and sham challenge. LPS has been shown to induce pulmonary and vascular hyperreactivity in mice [19]. In that study LPS had no effect on resistance. LPS has also been demonstrated to induce bronchial hyperresponsiveness in humans [20]. We found no increase in pulmonary resistance after LPS. This agrees with data from previous reports. In contrast the functional effects of TLR-2 agonists have not been characterized very well. To our knowledge this is the first study to investigate the effect of intratracheal PAM3 application on lung function parameters. At present it remains unclear how PAM3 augments pulmonary resistance. However, using more sophisticated analysis we found that PAM3 increased total resistance and tissue damping which represents tissue resistance. In contrast, no effect was observed in resistance of the central airways (Newtonian resistance). This finding suggests that PAM3 has an effect in the lung parenchyma and in the small airways but not in the central airways. In addition, only PAM3 challenge significantly decreased compliance and led to a significant increase in elastance.

## Conclusion

In conclusion in our study we found that both LPS and PAM3 can further increase ventilator-induced inflammation. In contrast to LPS PAM3 significantly increased resistance. The underlying mechanisms are under current investigation. It is tempting to speculate that bacterial infection of ventilated lungs amplifies VILI and that increased resistance due to PAM3 in case of Gram-positive bacterial infection may support bacterial colonization and airway obstruction. Future studies will have to further evaluate the effect of PAM3 on ventilated lungs and ventilator-associated inflammation.

## Competing interests

The authors declare that they have no competing interests.

## Authors' contributions

HPH and PZ designed the experimental protocol, did the data analysis and wrote the manuscript. HPH and DK performed the experiments. TG und EV did the pathologic analysis. All authors read and approved the final manuscript.

## Pre-publication history

The pre-publication history for this paper can be accessed here:

http://www.biomedcentral.com/1471-2466/10/20/prepub
